# Transcriptomic profile of tobacco in response to *Alternaria longipes* and *Alternaria alternata* infections

**DOI:** 10.1038/srep25635

**Published:** 2016-05-09

**Authors:** Shengchang Duan, Xiao Ma, Wei Chen, Wenting Wan, Yuqi He, Xiaoqin Ma, Yujin Ma, Ni Long, Yuntao Tan, Yangzi Wang, Yujie Hou, Yang Dong

**Affiliations:** 1 Faculty of Life Science and Technology, Kunming University of Science and Technology, Kunming, 650500, China; 2 Yunnan Agricultural University, Kunming, 650201, China; 3 Yunnan Research Institute for Local Plateau Agriculture and Industry, Kunming, 650201, China; 4 Public Technical Service Center, Kunming Institute of Zoology, Chinese Academy of Science, Kunming, 650223, China

## Abstract

Tobacco brown spot caused by *Alternaria* fungal species is one of the most damaging diseases, and results in significant yield losses. However, little is known about the systematic response of tobacco to this fungal infection. To fill this knowledge gap, *de novo* assemblies of tobacco leaf transcriptomes were obtained in cultivars V2 and NC89 after the inoculation of either *Alternaria longipes* (AL) or *Alternaria alternata* (AA) at three different time points. We studied the gene expression profile of each cultivar-pathogen combination, and identified eight differentially expressed genes shared among all combinations. Gene ontology enrichment analysis of the differentially expressed genes revealed key components during the fungal infection, which included regulation of gene expression (GO:0010468), regulation of RNA metabolic process (GO:0051252), tetrapyrrole binding (GO:0046906), and external encapsulating structure (GO:0030312). Further analyses of the continuously upregulated/downregulated genes and the resistance genes demonstrated that the gene expression profile upon fungal infection was contingent on the specific cultivar and pathogen. In conclusion, this study provides a solid foundation for the investigation of plant-pathogen interaction, and is of great importance for disease prevention and molecular breeding.

The model organism and widely cultivated non-food crop *Nicotiana tabacum* (common tobacco) is susceptible to numerous viral and fungal infections. These pathogens can cause destructive symptoms on the tobacco leaf, root and stem, i.e. anthracnose, black root rot, frogeye leaf spot, verticillium wilt and brown spot. Among these diseases, brown spot in particular incurs significant losses in many tobacco-planting areas in China every year^1^,^2^. Even though wide applications of fungicides and biological antagonists are major control measurements of brown spot in agriculture, they have provoked public concerns about health and environmental safety issues of the chemicals. In comparison, genetic manipulation is considered as one of the eco-friendly control methods to contain brown spot in the tobacco-planting areas^3^.

Two different species of the genus *Alternaria*, *A. longipes* (AL) and *A. alternata* (AA), are the major cause of brown spot in tobacco^1^,^2^,^4^,^5^,^6^,^7^. They produce a variety of toxic secondary metabolites that damage plant tissues and result in necrotic lesions. For instance, *A. longipes* has been reported to produce host-specific toxins to tobacco (AT-toxin), which play a role in pathogen invasion of the plant tissue. The infection of host plant primarily occurs in spring in warm weather, when the fungal spores germinate and directly penetrate into the lower leaves of tobacco. This results in the appearance of circular spots (0.635–3.175 cm in diameter) on the leaves^4^. As the plant continues to grow, fungal toxins not only causes infected leaves to age prematurely, but also causes bright yellow halo to appear around leaf spots. At the end of the tobacco harvesting, enormous amount of fungal spores were released into the air for the next cycle of infection. Additionally, the fungi can persist as mycelium in dead leaf tissue for several months.

The first line of defense against pathogenic microorganisms in plant is the recognition of pathogen-associated molecular patterns (i.e. bacterial flagellin, lipopolysaccharides) by plant transmembrane receptors that activates basal defense^8^. More importantly, plants also rely on the effector-triggered immunity to fight against pathogens that try to evade the first fine of defense^8^. The specificity determinants of effector-triggered immunity are encoded by resistance (R) genes^8^,^9^. The majority of R genes belong to the nucleotide-binding site leucine-rich repeat (NB-LRR) family, which encodes proteins with nucleotide binding site (NBS) and leucine-rich repeat (LRR) domains. The NBS domain primarily promotes the exchange of ADP for ATP and activates downstream signaling^8^. The LRR motifs appear to provide a versatile structural framework for the formation of protein-protein interactions^9^. These large, abundant R gene proteins are involved in the detection of diverse pathogens, including bacteria, viruses, fungi, nematodes, insects and oomycetes^10^. In addition, other functional tobacco genes have been implicated in the resistance to pathogens. For example, overexpression of GbERF2 transcription factor in tobacco can enhances resistance to brown spot^11^. Furthermore, many pathogenesis-related (PR) proteins have been reported to play important roles in the protection against fungal pathogens. These include plant chitinases, thaumatin-like proteins, ß-1,3-glucanase and the vast majority of wine proteins^12^,^13^.

As complicated as the mechanisms of disease resistance in plants can be, researchers believed that a global investigation of gene expression files during disease infections will help identify key components of resistance pathways^14^. So far, there has not been any report on the transcriptome of tobacco in response to *Alternaria* species infection. Two tobacco cultivars: *N. tabacum* L. cv. V2 (hereinafter referred as V2) and *N. tabacum* L. cv. NC89 (hereinafter referred as NC89), show different resistant to brown spot. Here, we performed RNA sequencing analysis of the gene expression profiles in both V2 and NC89 tobacco leaves, which were infected with either *A. longipes* or *A. alternata* at three time points: 6 h, 24 h and 72 h post inoculation. By comparing the gene expression patterns between V2 and NC89, we found some plant defense and disease resistance genes were differentially expressed between the two cultivars. The acquired transcriptome data provide an invaluable resource for understanding the response/resistance to *Alternaria* infection in tobacco.

## Results

### Early symptoms of brown spot on tobacco leaves

**Figure 1:**
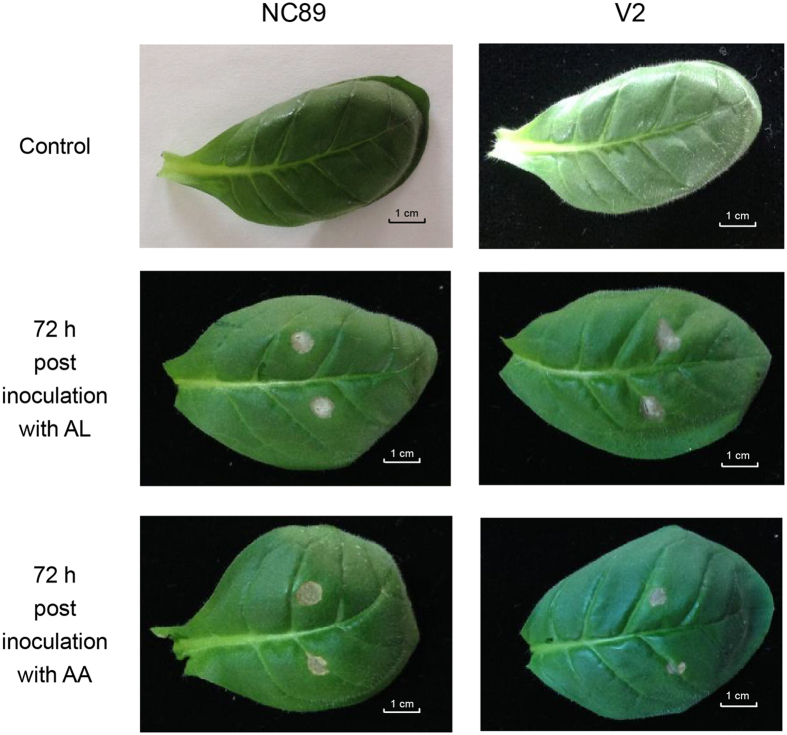
Symptoms of the brown spot. The characteristic necrosis symptom was visible on the infected tobacco leaves.

The early noticeable symptom appeared as a greyish spot of 0.3~0.4cm in diameter on the V2 and NC89 leaves about 72 h after inoculation with either AL or AA (Fig. 1). By direct observation, the AA-induced spots on the NC89 leaf were bigger than those on the V2 leaf. In comparison, the AL-induced spots were of similar size on the leaves of both cultivars. The symptoms were not apparent at 6 h and 24 h after inoculation.

### Sequencing and assembly of the tobacco leaf transcriptome

*N. tabacum* is an allotetraploid (2n = 48) plant. It is believed to have arisen from the hybridization of maternal donor *N. sylvestris* (2n = 24) and paternal donor *N. tomentosiformis* (2n = 24)^15^. Here, we *de novo* assembled a high quality tobacco transcriptome for further studies.

For cultivars V2 and NC89, seven sequencing libraries were generated for each from the total RNA of (1) healthy leave tissues, (2) infected leave tissues by AA for 6, 24 and 72 h, and (3) infected leave tissues by AL for 6, 24 and 72 h (Table 1). Illumina high-throughput second generation sequencing produced about 332.2 million raw reads for V2, and about 625.7 million for NC89. After the removal of adapter-containing reads, poly-N-containing reads and low quality reads from the raw data, about 276.5 million clean reads were obtained for V2, and about 531.4 million for NC89. All 807.9 million clean reads from V2 and NC89 were used for the transcriptome assembly. Initial assembly using Trinity^16^ yielded 391,676 contigs with a N50 value of 993 bp. Further prediction of open reading frames using TransDecoder obtained 31,572 unigenes, contained 64,184 transcripts, with a N50 value of 2,053 bp and GC content of 40.65% (Table 2).

To evaluate the reliability of the assembly, we mapped the clean reads from the initial V2 and NC89 libraries back to the Trinity assembled transcriptome. In the end, 74.9% left reads and 74.1% right reads could be map back to the assembled transcriptome, respectively. The concordant pair alignment rate was 66.4% (Table 3). These results demonstrated the high quality of the transcriptome assembly.

We performed gene function annotation analysis of the 31,572 unigenes. Using Blastp with a cutoff E-value of 10^−5^, we annotated 27,318 unigenes against the NR database, 20,559 unigenes against Swiss-Prot database, and 16,370 against KEGG database. In total, 27,348 unigenes had at least one homologous counterpart from these databases (Supplementary Table 1). The annotation against NR database was preferentially used in subsequent data analysis.

### Differentially expressed genes for each cultivar-pathogen combination at three time points after infection

The FPKM value for each unigene in all 14 libraries was computed and shown in Supplementary Table 2.The gene expression profiles in healthy V2 and NC89 leaves were used as baselines, respectively. If the gene expression in infected leaves recorded a 2-fold (or more) difference relative to baseline (*p*-value < 0.05), this gene was regarded as the differentially expressed gene (DEG).

**Figure 2:**
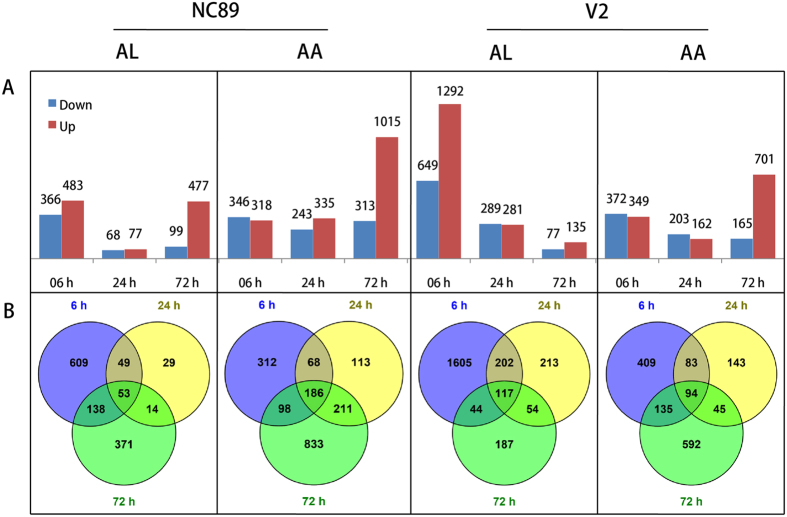
Summary for the differentially expressed genes. (**A**) Statistics of the number of differentially expressed genes in four cultivar-pathogen combinations. (**B**) Venn diagram showing the number of specific and common differentially expressed genes in four cultivar-pathogen combinations.

For NC89 inoculated with AL, there were 849 DEGs at 6 h, and just 145 DEGs at 24 h after inoculation. The number of DEGs at 72 h after inoculation rose to 576 due to a marked increase of upregulated DEGs (Fig. 2A, first panel). In comparison, AA-infected NC89 leaves recorded 664, 578 and 1,328 DEGs at those three different time points, respectively. In particular, the number of upregulated DEGs at 72 h after inoculation was 1,015, much larger than the numbers of DEGs in other groups (Fig. 2A, second panel). For V2 inoculated with AL, the number of DEGs decreased from 1,941 at 6 h after inoculation, to 570 at 24 h after inoculation, and finally to 212 at 72 h after inoculation. The number of upregulated DEGs was larger than that of downregulated DEGs at 6 h (1,292 vs. 649 genes) and 72 (135 vs. 77 genes) after inoculation (Fig. 2A, third panel). For V2 inoculated with AA, there were 721, 365 and 866 DEGs at 6 h, 24 h and 72 h after inoculation, respectively. The number of downregulated genes was slightly bigger than the number of upregulated genes at 6 h and 24 h after inoculation, but much smaller than the number of upregulated genes at 72 h after inoculation (Fig. 2A, fourth panel).

Corresponding to each above mentioned cultivar-pathogen combination, a Venn diagram was generated from the DEG lists at 6 h, 24 h and 72 h after inoculation to identify shared members. As shown in Fig. 2B, there were 53 genes that were differentially expressed at all three time points in AL-infected NC89, and 186 genes in AA-infected NC89. For AL-infected V2, there were 117 DEGs that could be identified at all three time points. And for AA-infected V2, 94 genes were common DEGs at all three time points.

### Shared differentially expressed genes during *Alternaria* infection

**Figure 3:**
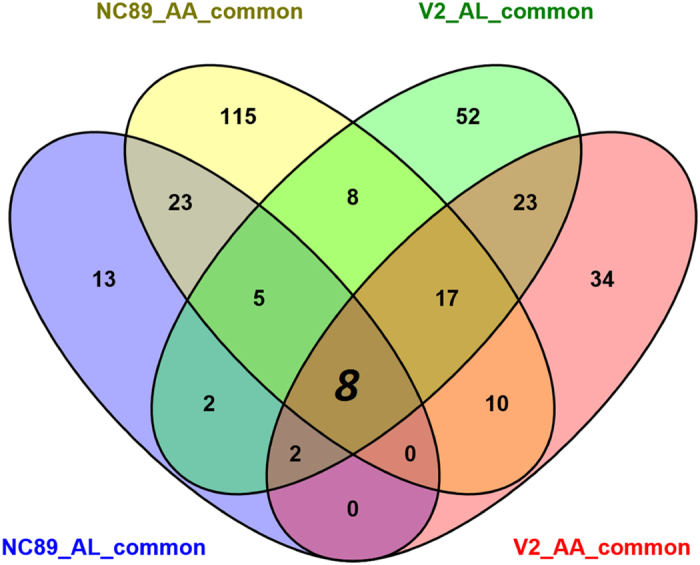
Venn diagram showing the commonalities and differences in four lists of shared differentially expressed genes.

The above mentioned four lists of shared DEGs were further analyzed to find commonalities and differences. As shown in the Venn diagram of Fig. 3, there were eight common DEGs in all four lists of DEGs (Supplementary Table 3). Two genes, c1013452_g1 and c1021713_g1, were predicted to encode putative peroxidases. Plant peroxidases are known to participate in defense against arthropods and pathogens^17^. In line with this, both genes were significantly upregulated in NC89 and V2 after the inoculation of either AA or AL. The gene c1032484_g1, which encodes a glucan endo-1,3-beta-glucosidase, was significantly downregulated in NC89 and V2 after the inoculation of either AA or AL. Tobacco basic beta-l,3-glucanase was a major soluble protein component of the cultured tobacco cells, and it was implicated in the defense response of plants against potential pathogens^18^. The branched-chain amino acid aminotransferase gene (c1034473_g6) was also significantly downregulated in NC89 and V2 after the inoculation of either AA or AL. Amino acid aminotransferase helps catabolize branched-chain amino acids, aromatic amino acids and aspartic acid^19^. It is possible that inhibition of branched-chain amino acid catabolism is important for the response to *Alternaria* infection. The expression of malate synthase gene (c1112440_g1) was significantly downregulated in NC89 and V2 after the inoculation of either AA or AL. Malate synthase, which helps synthesize malate from glyoxylate and acetyl-CoA, was shown to play important roles in plant pathogenesis^20^. The functions of three other DEGs were not clear.

Additionally, different numbers of unique DEGs were found for each cultivar-pathogen combination: 13 unique DEGs in NC89-AL, 115 unique DEGs in NC89-AA, 52 unique DEGs in V2-AL, and 34 unique DEGs in V2-AL.

### Gene ontology (GO) enrichment analysis of differentially expressed genes

Within 31,572 identified unigenes, the protein products of 11,730 unigenes were annotated with at least one GO term (Supplementary Table 4). Using these 11,730 unigenes as references, 609 out of 1,263 DEGs in NC89-AL, and 930 out of 2,422 DEGs in V2-AL, were both enriched in 25 GO terms. Similarly, 871 of 1,821 DEGs in NC89-AA and 725 out of 1,501 DEGs in V2-AA, were enriched in 20 and 21 GO terms, respectively (Supplementary Table 5).

There were four enriched GO terms in NC89-AL that could also be found in V2-AL. These terms included regulation of gene expression (GO:0010468), regulation of RNA metabolic process (GO:0051252), tetrapyrrole binding (GO:0046906), and external encapsulating structure (GO:0030312). The majority of the NC89-AL and V2-AL specific biological processes GO terms were mainly related to the metabolic and biosynthetic processes. The GO term photosystem (GO:0009521) was only enriched in V2-AL with 13 associated genes. Further examination of the transcriptome data showed that 11 of these 13 genes had lower expression levels at the three time points after inoculation relative to controls.

In the case of AA infection, the majority of enriched GO terms in NC89-AA could also be found in V2-AA. These terms included 11 GO terms in the biological processes category, one GO term in the molecular function category, and two GO terms in the cellular component category. Correspondingly, only six and seven GO terms were specifically enriched in NC89-AA and V2-AA, respectively.

It is worth noting that three GO terms, regulation of gene expression (GO:0010468), regulation of RNA metabolic process (GO:0051252), and external encapsulating structure (GO:0030312), were found in all four cultivar-pathogen combinations.

### Continuously upregulated and downregulated genes during *Alternaria* infection

Continuously upregulated genes during the course of *Alternaria* infection were screened out if the FPKM value of a gene at any time point was at least 1.5-fold of the FPKM value at the previous time point. This screening criterion resulted in 47 continuously upregulated genes in NC89-AL and V2-AL, respectively (Supplementary Table 6). Among these genes, c1134946_g1 was identified in both NC89-AL and V2-AL. The gene c1134946_g1 encodes a putative wound-responsive protein that is rich in lysine (K), glutamic acid (E) and aspartic acid (D)^21^. Its expression at 72 h in NC89-AL and V2-AL were almost 35-fold and 54-fold of the expressions at 0 h, respectively. The screening criterion also identified 427 continuously upregulated genes in NC89-AA, and 30 continuously upregulated genes in V2-AA (Supplementary Table 6). These two pathogen-cultivar combinations shared four continuously upregulated genes: the serine/threonine-protein kinase gene (c1042136_g2), allene oxide synthase gene (c1026266_g2), two-component response regulator ARR9-like gene (c1029343_g3) and probable iron/ascorbate oxidoreductase gene (c1027160_g2).

Continuously downregulated genes during the course of *Alternaria* infection were screened out if the FPKM value of a gene at any time point was at least 1.5-fold of the FPKM value at the next time point. This screening criterion resulted in 6, 17, 46, and 13 continuously downregulated genes in NC89-AL, V2-AL, NC89-AA and V2-AA, respectively (Supplementary Table 6). No shared genes were found between NC89-AL and V2-AL, or between NC89-AA and V2-AA.

### Analysis of the NBS gene families during *Alternaria* infection

We identified 144 candidate NBS genes from the transcriptome (Supplementary Table 7). These candidate genes included 13 TOLL/interleukin-1 receptor (TIR) type NBS genes, and 131 non-TIR type NBS genes. Within TIR type NBS genes, the protein products of six genes do not have LRR domains. Within the non-TIR type NBS genes, there were 30 genes whose protein products have a coiled-coil (CC) N-terminal domain, six genes whose protein products include the DUF3542 N-terminal domain, two genes whose protein products include the RPW8 N-terminal domain.

**Figure 4:**
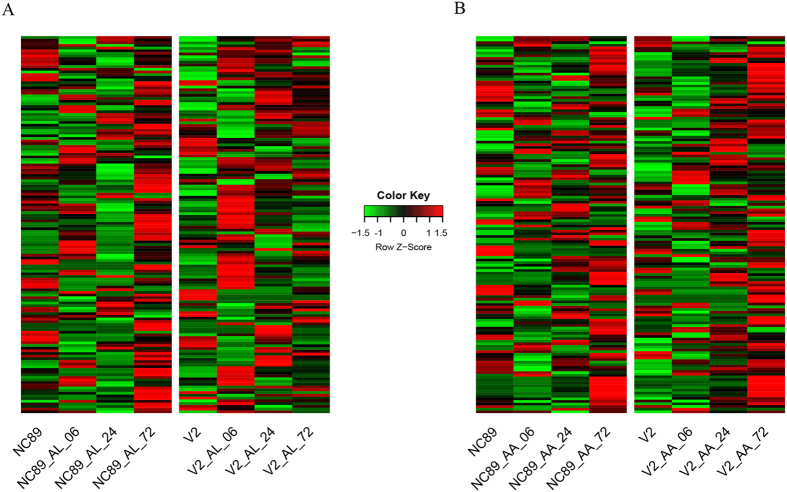
Heat-map depicting the changes of NBS genes expression in cultivar-pathogen combinations.

The majority of NBS genes were significantly upregulated in NC89-AL, NC89-AA and V2-AA at 72 h (Fig. 4). In comparison, the majority of NBS genes were significantly upregulated in V2-AL at 6 h (Fig. 4), displaying a distinct expression pattern. For example, the expression levels of c1042342_g1 (disease resistance protein RPS2) and c1041513_g1 (disease resistance RPP13-like protein) were high at 72 h in NC89-AL, NC89-AA, and V2-AA, but not so distinct in V2-AL at the same time point. Additionally, some NBS genes (i.e. c1135034_g1, c1043786_g1) were significantly upregulated in V2-AL and V2-AA during the course of the infection. But in NC89-AL and NC89-AA, the expression of these genes was downregulated at 6 h before subsequent upregulation at 24 h and/or 72 h.

### Quantitative RT-PCR (qRT-PCR) validation of differentially expressed genes

A subset of seven genes, which responded to *Alternaria* infection, was selected for quantitative real-time PCR (qRT-PCR) analyses (Supplementary Figure 1). The qRT-PCR analyses showed the trends of expression were consistent with those found by RNA-Seq.

## Discussion

We assembled and analyzed the leaf transcriptomes of two tobacco cultivars NC89 and V2 after the inoculation of *Alternaria* pathogens. To our knowledge, this study is the first large-scale investigation of gene expression patterns with regard to tobacco-*Alternaria* interaction. The results will assist in the discovery and annotation of important genes in plant defense response, physiology and metabolism.

In the event of pathogen invasion, the biological process to reinforce cell wall is usually rapidly activated, leading to increased disease resistance in plants^22^. Indeed in the current study, the GO term external encapsulating structure (GO:0030312) was enriched for the DEGs of all four cultivar-pathogen combinations. This result suggests that active reorganization/modification of cell walls may be a common mechanism for the tobacco plants to fight against *Alternaria* infection. Despite this commonality, the expressions of some cell wall modification genes (i.e. expansin-like B1-like gene) were differently regulated in each cultivar-pathogen combination. Further investigation of these genes will provide valuable molecular information on the host susceptibility to different *Alternaria* strains.

It is worth mentioning that various numbers of unique GO terms were enriched in each cultivar-pathogen combination. For example, the cellular component GO terms thylakoid (GO:0009579) and thylakoid part (GO:0044436) were only enriched in V2-AL. Close examination of these GO enrichment may provide molecular insight into the phenotypic variations of response to pathogens in different cultivars. In tobacco, cucumber mosaic virus R46C mutants can repress the expression of photosynthesis-related genes, and cause chloroplast abnormalities with fewer thylakoid membranes^23^. Given those unique GO terms, it is therefore very likely that V2-AL could be linked to chloroplast abnormalities, and the corresponding DEGs may be good research candidates for such phenotypes.

The differential regulation of gene expression was also apparent between cultivars. Take *HARPIN-INDUCED 1* (*HIN1*) for example, the expression of *HIN1* was strongly upregulated during leaf- and flower-senescence after TMV infection in tobacco^24^. Similar upregulation of the gene (c1033713_g2) was identified in NC89-AL, but not so distinct in V2-AL. Additionally, *POLYPHENOL OXIDASE* (*PPO*) is proposed to be involved in plant defense against pathogens^25^. The expression level of *PPO* transcript (c1039691_g9) was high in V2-AL, but not in NC89-AL. Further investigation of these differentially regulated genes will as well provide insight into the different pathogen responses in different tobacco cultivars.

The transcriptome data obtained in this study is also a great resource for cross-checking genes that were previously identified in the plant-pathogen interaction process. For instance, rapid accumulation followed by a rapid decrease of *KED* gene transcripts was shown within 1 h after wound initiation in tobacco^21^. *KED* activation, signaled by cytosolic acidification, was reported to induce the expression of defense genes^21^. In this study, the expression of a *KED* gene (c1134946_g1) was continuously upregulated for 72 h after AL infection, showing its unique role during fungal infection. In addition, the expressions of two *GLUTATHIONE S-TRANSFERASES* were significantly increased in *Nicotiana benthamiana* following the infection by *Colletotrichum destructivum* and *C. orbiculare*^26^. However, this study showed that the expressions of a *GLUTATHIONE S-TRANSFERASE* gene (c1157960_g1) in both cultivars were significantly reduced after AL infection. This discrepancy suggests that certain genes may play different roles during the plant-pathogen interaction in different plant species.

In conclusion, the leaf gene expression patterns of NC89 and V2 after *Alternaria* infection provide a solid foundation for future studies of the molecular mechanisms underlying tobacco brown spot response.

## Materials and Methods

### Plant material and RNA isolation

The *N. tabacum* L. cv. NC89 is sensitive to the brown spot, and the *N. tabacum* L. cv. V2 was obtained the resistance to brown spot disease by inducing from NC89 with the hypovirulent viruses CMV-SV52^27^. Tobacco seeds (V2 and NC89) were sterilized using chlorine gas and grown axenically on MS medium^28^ for 2 months under a controlled16h-light/8h-dark cycle. After 15 days of growth on the potato dextrose agar media, the conidia of *A. longipes* (AL) and *A. alternata* (AA) were harvested to prepare suspensions with a concentration of 1 × 10^6^ conidia/ml. 5 μl conidial suspension was used to inoculate the leaf from the aseptic seedlings of V2 and NC89. Two cultivars and two *Alternaria* strains yield four cultivar-pathogen combinations: V2_AL, V2_AA, NC89_AL and NC89_AA. Every combination was harvested at three time points, that is, 6, 24, and 72 hours post inoculation, respectively. For the 6 and 24 time points, inoculated plants were kept for 72 hours after harvest to monitor the success of the inoculations. Leaf tissues from healthy aseptic tobacco seedling were the controls for the experiment.

Total RNA was isolated with TRIzol (Invitrogen) from each sample according to the manufacturer’s instructions. It was treated with RNase-free DNase I (Invitrogen) for 30 min at 37 °C to remove residual DNA. The RNA quantity and quality were determined using Agilent Technologies 2100 Bioanalyzer (Agilent).

### cDNA library preparation and sequencing

3 μg of total RNA per sample was used as input material for the RNA sample preparation. Beads with oligo (dT) were used to isolate poly(A) mRNA from total RNA. RNA sequencing libraries were constructed from these mRNA using the TruSeq RNA Sample Preparation Kit (Illumina, San Diego, USA). Briefly, the Elution 2-Frag-Prime (94 °C for 8 minutes, 4 °C hold) was used to elute, fragment and prime the mRNA with Elute, Prime, Fragment Mix (Illumina). First strand cDNA synthesis was performed with First Strand Master Mix and SuperScript II mix (ratio: 1ul SuperScript II/7ul First Strand Master Mix) (Invitrogen). The second strand was synthesized with Second Strand Master Mix (Illumina) and Ampure XP beads (Illumina) were used to separate the double-stranded (ds) cDNA from the 2nd strand reaction mix. After end repair and the addition of a 3′-dA overhang, the cDNA was ligated to Illumina PE adapter oligo mix (Illumina), and size-selected for 350 ± 20 bp fragments by gel purification. After 15 cycles of PCR amplification, the 350 bp paired-end libraries were sequenced using the paired-end sequencing module (100 bp at each end) of the Illumina HiSeq 2000 platform.

### *De novo* transcriptome assembly

Raw image data were transformed by base calling into sequence data. These raw reads were stored in fastq format, and processed through Trimmomatic (Version 0.32)^29^. This step removed reads containing adapter, reads containing poly-N and low quality reads from the raw data and yielded clean data for downstream analyses. *De novo* assembly of transcriptome was carried out with a short reads assembling program, Trinity (Version: trinityrnaseq_r20140717)^30^. This resulted in a set of sequences, which could not be extended on either end. The candidates which have most probable longest-ORF were generated from the Trinity assembly result using TransDecoder (https://transdecoder.github.io/) and manual review. A set of thus candidates (“mRNA” sequences) was used for reference transcriptome. If multiple transcripts belong to a unigene, the coding sequences of a transcript was extracted and used for functional annotations of the unigene.

### Gene annotation

Protein sequences corresponding to the coding sequences of unigenes were obtained, and then searched against NCBI non-redundant (nr) database, Swiss-Prot and KEGG databases using Blastp with a cut-off E-value of 10^−5^.

### Quantification of gene expression level

RSEM^31^ was used for transcript abundance estimation and expression value computation. To estimate transcript abundance, the original reads were aligned to the transcriptome using bowtie2^32^. Then, RSEM was executed to estimate expression values based on the resulting alignments. Simultaneously, the fragments per kilobase of exon per million fragments mapped (FPKM) value of each unigene was calculated based on the length of the gene and mapped reads count.

### Differential expression analysis

The detection of differentially expressed genes was performed using edgeR program package^33^ with the rigorous algorithm method. The threshold of *p*-value in multiple test and analysis was determined by false discovery rate (FDR). The differentially expressed genes were deemed significant by the following criteria: FDR < 0.05 and the absolute value of Log_2_ (Ratio) ≥1.

Especially, continuously upregulated genes during the course of *Alternaria* infection were screened out if the FPKM value of a gene at any time point was at least 1.5-fold of the FPKM value at the previous time point.

### GO enrichment analysis

GO annotation of predicted protein sequences were acquired using InterProScan-5.8–49.0^34^. The GO enrichment was done with Ontologizer 2.0^35^ by using one-sided Fisher’s exact test, the Parent-Child-Union method, and the multiple-testing correction method was set to “Westfall and Young single-step”, with a p-value cut-off of 0.001 (adjusted p-value using multiple-testing correction method (p.adjusted)). All genes with GO annotation were used as reference, and all DEGs with GO annotated of each cultivar-pathogen combination were used as study set, respectively.

### Identification of the NBS gene family

Predicted protein sequences from the unigenes were scanned by HMMER (Version 3.0)^36^ with the Hidden Markov Model (HMM) against the Pfam^37^ NBS (NB-ARC) family (PF00931). A possible tobacco protein set (E-value < 10) was aligned against Pfam database to verify the HMMER result. The sequences that had no NBS domains were discarded. The NBS domain and other domains were synthesized in these proteins with E-value < 10^−5^. Because coiled-coil domains could not be identified through the Pfam method, we used COILS software (Version 2.2)^38^ to predict the coiled-coil domains with a P score cut-off of 0.05.

### Quantitative RT-PCR

In order to validate the RNA-Seq results, qRT-PCR analysis was performed using 12 RNA samples (Supplementary Figure 1), which were used in the sequencing libraries preparation. Reverse-transcription was performed using total RNAs (1 μg) with PrimeScript^TM^ RT reagent Kit (TaKaRa) according to the manufacturer’s protocol. The primer sets were designed using Oligo 6.44 software within the CDS region of nucleotide sequences (Supplementary Table 8), and the parallel reactions using the *actin* (NCBI Accession number: JQ256516) primers were performed to normalize the amount of template cDNA added to each reaction. The gene specific primers were diluted to a final concentration of 10 μM. For each sample 30 ng of cDNAs were used as template in 20 μl PCR reactions containing 10 μl SYBR Premix Ex Taq II (Tli RNaseH Plus) (2x) (TaKaRa) and 0.8 μl forward and 0.8 μl reverse primers. PCR reactions were performed on a QuantStudio 12K Flex real-time PCR system (Life Technologies) as follows: denaturation at 95 °C for 3 min, followed by 40 cycles at 95 °C for 20 s, 60 °C for 20 s, and 72 °C for 30 s. Fluorescent intensity data were acquired during the 72 °C extension step. At the end of the amplification cycles, a melting curve step was performed to check for specificity of the amplified product, which were run at 95 °C for 15 s and 60 °C for 1 min followed by an increase in temperature from 60 to 95 °C (0.05 °C/s) with continuous fluorescence recording. All experiments were used in quadruplicate for each sample and relative gene expression levels were calculated using the 2^−ΔΔCT^ method^39^.

## Additional Information

**Data availability**: The RNA-Seq raw data were deposited in the NCBI Sequence Read Archive (SRA) with the accession number SRX1048180 and SRX1406790

**How to cite this article**: Duan, S. *et al.* Transcriptomic profile of tobacco in response to *Alternaria longipes* and *Alternaria alternata* infections. *Sci. Rep.* **6**, 25635; doi: 10.1038/srep25635 (2016).

## Supplementary Material

Supplementary Information

Supplementary Table 1

Supplementary Table 2

Supplementary Table 3

Supplementary Table 4

Supplementary Table 5

Supplementary Table 6

Supplementary Table 7

Supplementary Table 8

## Figures and Tables

**Table 1:** Statistics of Illumina sequencing data.

Sample	Raw read length (bp)	Raw read numbers	Raw data bases (Gb)	Clean read numbers	Clean data bases (Gb)
V2	100	51,627,124	5.163	41,800,428	4.180
V2_AL_06	100	45,431,094	4.543	41,802,402	4.180
V2_AL _24	100	47,412,962	4.741	37,235,258	3.724
V2_AL _72	100	36,315,648	3.632	33,489,918	3.349
V2_AA _06	100	16,681,012	1.668	11,887,184	1.189
V2_AA _24	100	63,463,382	6.346	50,457,162	5.046
V2_AA _72	100	71,339,808	7.134	59,922,442	5.992
NC89	100	32,050,662	3.205	29,124,816	2.912
NC89_AL_06	100	46,344,166	4.634	43,033,824	4.303
NC89_AL _24	100	14,971,724	1.497	12,220,324	1.222
NC89_AL _72	100	81,726,554	8.173	71,262,904	7.126
NC89_AA _06	100	125,912,182	12.591	113,830,604	11.383
NC89_AA _24	100	102,220,638	10.222	81,754,766	8.175
NC89_AA _72	100	222,544,794	22.254	180,174,190	18.017
Total		958,041,750	95.803	807,996,222	80.798

**Table 2:** Summary of the assembly results.

Trinity contig numbers	Trinity assembled bases (bp)	Trinity contig N50 (bp)	Final transcript numbers	Final percent GC	Final transcript N50 (bp)	Final assembled bases (bp)	Final unigene numbers
391,676	237,828,789	993	64,184	40.65%	2,053	103,504,886	31,572

**Table 3:** Summary for clean reads mapping to Trinity assembled transcriptome.

	Input	Mapped	Multiple alignments	Mapped rate of input
Left reads	403,998,111	302,758,273	113,584,692	74.9%
Right reads	403,998,111	299,531,909	112,379,840	74.1%
Aligned pairs	403,998,111	268,929,138	103,395,229	66.4%
