# Supplementary material for: Transcriptomic profile of tobacco in response to Alternaria longipes and Alternaria alternata infections
Source: Sci Rep. 2016 May 9;6:25635. doi: 10.1038/srep25635 (PMC4860569; doi:10.1038/srep25635)
Supplement: Supplementary Information [file srep25635-s1.pdf]

# Transcriptomic profile of tobacco in response to *Alternaria longipes* and *Alternaria alternata* infections

Shengchang Duan, Xiao Ma, Wei Chen, Wenting Wan, Yuqi He, Xiaoqin Ma, Yujin Ma, Ni Long, Yuntao Tan, Yangzi Wang, Yujie Hou, Yang Dong

**Supplementary Table 1** Unigenes annotation.

**Supplementary Table 2** FPKM values of unigenes.

**Supplementary Table 3** Expression and annotation of eight shared differentially expressed genes during *Alternaria* infection.

**Supplementary Table 4** GO annotation for unigenes.

**Supplementary Table 5** GO enrichment for differentially expressed genes in NC89-AL, V2-AL, NC89-AA and V2-AA.

**Supplementary Table 6** List of continuously upregulated and downregulated genes during *Alternaria* infection.

**Supplementary Table 7** List of the predicted NBS proteins.

**Supplementary Table 8** List of oligonucleotide primers used for qRT-PCR experiments.

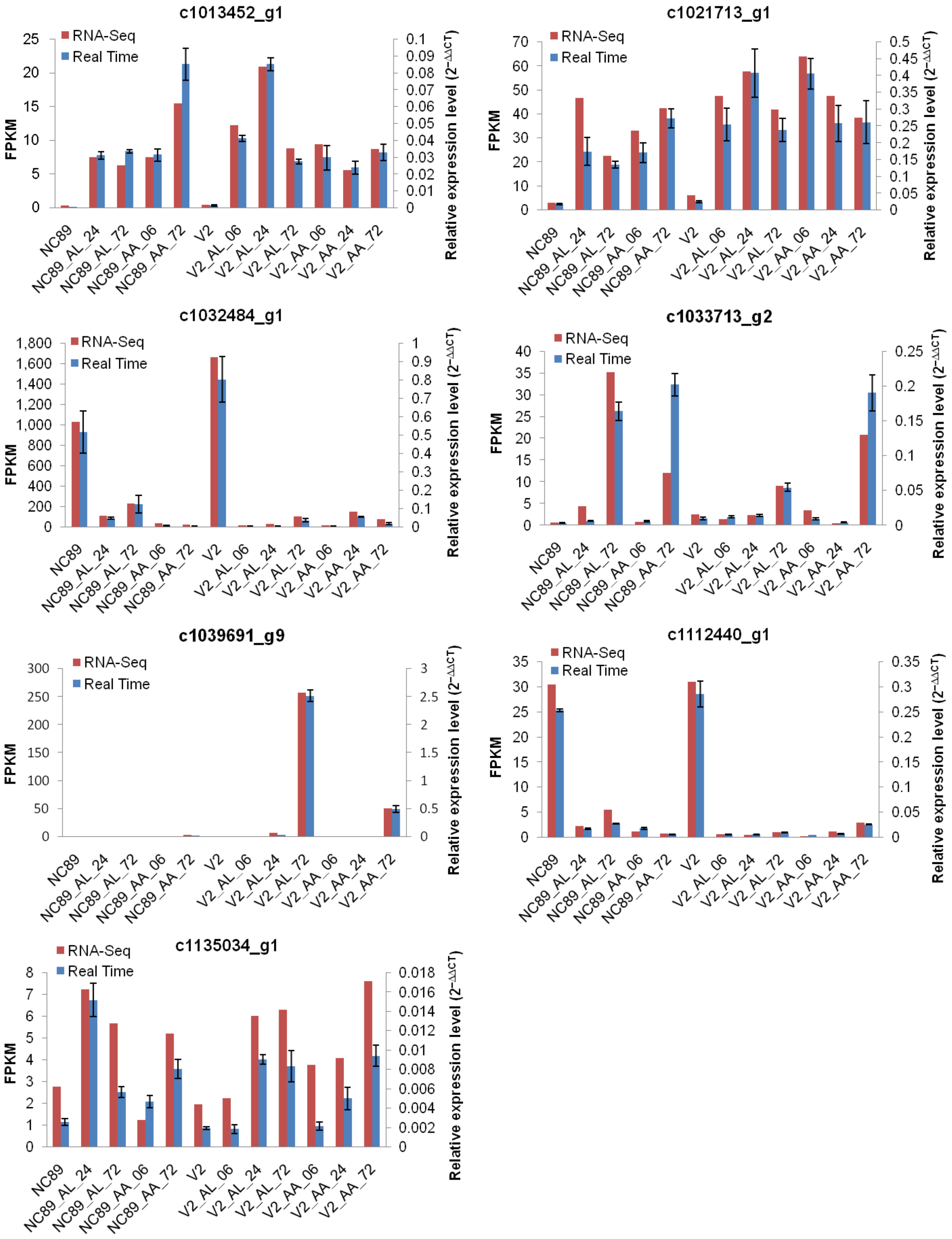
c1013452_g1
c1021713_g1
c1032484_g1
c1033713_g2
c1039691_g9
c1112440_g1
c1135034_g1
RNA-Seq
Real Time
FPKM
Relative expression level (2^−ΔΔCT)
NC89
NC89_AL_24
NC89_AL_72
NC89_AA_06
NC89_AA_72
V2
V2_AL_06
V2_AL_24
V2_AL_72
V2_AA_06
V2_AA_24
V2_AA_72

**Supplementary Figure 1 Quantitative RT-PCR (qRT-PCR) validation of the relative expression levels of genes selected from the DGE analysis.** Relative gene expression levels of selected genes were determined by RNA-Seq (Red) and qRT-PCR (Blue). The gene for *actin* was used as control for normalization of qRT-PCR analysis. The y-axis shows the normalized expression level of the genes. The x-axis indicates the control samples and tobacco-*Alternaria* combinations.
